# The distribution of the preferred directions of the ON–OFF direction selective ganglion cells in the rabbit retina requires refinement after eye opening

**DOI:** 10.1002/phy2.13

**Published:** 2013-06-26

**Authors:** Ya-Chien Chan, Chuan-Chin Chiao

**Affiliations:** 1 Institute of Molecular Medicine, National Tsing Hua UniversityHsinchu, 30013, Taiwan; 2 Institute of Systems Neuroscience, National Tsing Hua UniversityHsinchu, 30013, Taiwan; 3 Department of Life Science, National Tsing Hua UniversityHsinchu, 30013, Taiwan

**Keywords:** Direction tuning strength, retinal ganglion cells, tracer coupling pattern

## Abstract

The ON–OFF direction selective ganglion cells (DSGCs) in the mammalian retina respond differentially for an object moving in different directions. DSGCs can be further segregated into four functional subtypes, namely those responsible for the detection of motion in the superior, inferior, anterior, and posterior directions of the visual field. Although it has been known that the basic neural circuit of direction selectivity is established at around the time of eye opening, it is less known if the four DSGC subtypes can be unambiguously distinguished at this time and whether their preferred directions are aligned with four canonical axes at this developmental stage. By examining the preferred directions of DSGCs in P10-12 rabbit retinas and characterizing their distribution pattern, we have shown that the preferred directions of DSGCs at around the time of eye opening are not distinctly segregated but rather are diffusely distributed along the four canonical axes. Similar results were found in the mouse retina by reanalyzing previously published data. Furthermore, taking into account the fact that the direction tuning strength of DSGCs at P10-12 is weaker than that in adults, this was found not to be correlated with their preferred directions, which suggests that the maturations of direction selectivity and preferred direction are independent processes. In addition, we also found that the subtypes of DSGCs, which do not display tracer coupling pattern in the adult, show extensive coupling at P10-12. Taken together, the present study supports that the significant refinement after eye opening is required for the development of the four functional DSGC subtypes in the rabbit retina.

## Introduction

Direction selectivity is essential throughout the visual system (Hubel and Wiesel 1962; Barlow and Hill 1963; Levick et al. 1969; Bousfield 1977, 1978; Baker et al. 1981). Object motion in the preferred direction of these visual neurons evokes vigorous responses. In contrast, the same cells respond weakly to motion in their opposite, or null direction. In the rabbit retina, direction selective ganglion cells (DSGCs) fall into two types, ON and ON–OFF (Barlow et al. 1964), whereas in the mouse retina, there are three types, ON, OFF, and ON–OFF (Weng et al. 2005; Sun et al. 2006; Kim et al. 2008). Among them, the ON–OFF type of DSGCs is the most well-characterized type (Demb 2007; Zhou and Lee 2008; Elstrott and Feller 2009; Borst and Euler 2011; Wei and Feller 2011; Vaney et al. 2012). According to their preferred directions, ON–OFF DSGCs (referred as DSGCs hereafter) can be further segregated into four functionally different subtypes (Oyster and Barlow 1967; Oyster 1968) that are responsible for detection of the superior, inferior, anterior, and posterior motion directions across the visual field. During central projection, DSGCs send directional information to many brain areas, including the lateral geniculate nucleus (LGN), superior colliculus, nucleus of optic tract, medial terminal nucleus, and zona incerta (Stewart et al. 1971; Vaney et al. 1981; Pu and Amthor 1990; Huberman et al. 2009; Kay et al. 2011; Rivlin-Etzion et al. 2011). Thus, the outputs of these DSGCs may have a diverse variety of functions including motion perception and eye movement.

Accumulated evidence has shown that DSGCs exhibit direction selectivity at around the time of eye opening (Bowe-Anders et al. 1975; Masland 1977; Zhou and Lee 2005; Chan and Chiao 2008; Elstrott et al. 2008; Chen et al. 2009; Sun et al. 2011; Wei et al. 2011). It is important to know whether the functional organization of DSGCs in their four distinctly distributed preferred directions is also established at around the time of eye opening. Although a previous study in the mouse retina suggested that DSGCs seem to have four subtypes after eye opening (Elstrott et al. 2008), the distribution of their preferred directions in the young mouse retinas does not appear to be as distinctive as those found in adult mouse retinas. In order to further examine the functional organization of DSGCs at around the time of eye opening, we have characterized the distribution of the preferred directions of DSGCs using both cluster analysis and Fourier analysis in rabbits, a classical animal model for studying direction selectivity. We have also performed the same two analyses on the previous data in mice. In addition, it has been observed that the directional tuning strengths of DSGCs at around the time of eye opening in both rabbit and mouse retinas are much variable than those found in adult retinas (Chan and Chiao 2008; Elstrott et al. 2008), although the exception exists (Chen et al. 2009). Nevertheless, this suggests that some DSGCs may undergo further sharpening of their directional tuning after eye opening. To investigate if sharpening of the directional tuning of DSGCs and their preferred direction refinement involve a common developmental process, we have also examined the correlation between these two features at around the time of eye opening.

It was recently shown that only the superior subtype of DSGCs displays tracer coupling pattern in the adult rabbit retina (Kanjhan and Vaney 2008). However, it has been found that DSGCs are coupled to each other at the time of birth and decreased their coupling strength dramatically during development (DeBoer and Vaney 2005). It has been speculated that this decoupling of DSGCs may play a role in establishing their circuitry. As the percentage of tracer-coupled DSGCs at around the time of eye opening is still higher than those found in adult (DeBoer and Vaney 2005), this suggests that the superior subtype of DSGCs may not be the only one that remains tracer coupled at eye opening. Thus, in order to answer this question, we have also attempted to characterize the preferred directions of the DSGCs that still have tracer coupling at around the time of eye opening.

In this study, we provide evidence showing that the distribution of the preferred directions of DSGCs in both rabbits and mice at around the time of eye opening are not distinctly segregated but rather are diffusely distributed across the four canonical axes. Furthermore, the distribution of the preferred directions does not correlate to their tuning strength in terms of direction selectivity, which suggests that the developments of preferred direction and of directional tuning may be part of different processes. In addition, we have found that DSGCs with tracer coupling patterns are not confined to one subtype at around the time of eye opening. Therefore, our results suggest that refinement of all four functional subtypes of DSGCs is required after eye opening.

## Methods

### Ethical approval

All procedures were approved by the institutional animal care and use committee and were in accordance with the Association for Research in Vision and Ophthalmology Statement for the Use of Animals in Ophthalmic and Visual Research.

### Retina preparation

**Figure 1:**
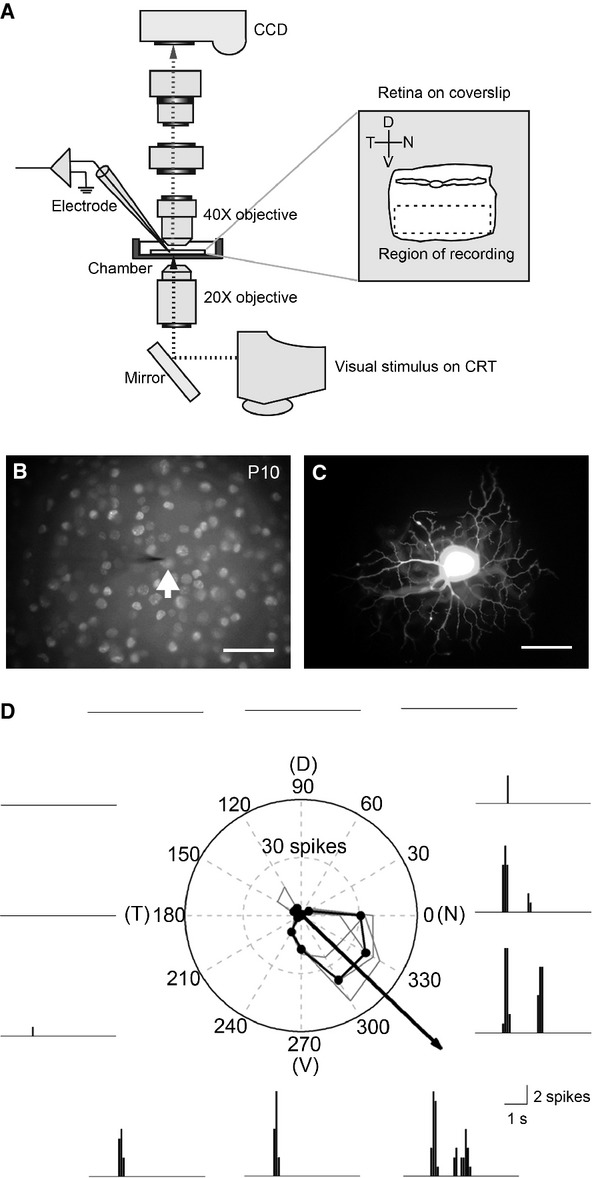
The preferred direction of a DSGC recorded at around the time of eye opening does not belong to one of four canonical directions. (A) A diagram of the recording setup. The enlarged area shows the orientation of whole-mount retina adhered onto a coverslip during extracellular recording. (B) A microphotograph of DAPI stained cell nuclei in the ganglion cell layer of a P10 rabbit retina. The arrow indicates the electrode tip and recorded cell nucleus. (C) A microphotograph of the targeted cell in (B) filled with Lucifer Yellow after recording, showing its dendritic morphology in the sublaminar a of the inner plexiform layer. (D) A polar plot of direction tuning from the recorded cell in (B). The black polygon is the average response of four trials, and the arrow is its vector sum, which represents the preferred direction. The representative peristimulus time histograms (the second trial) are also depicted around this polar plot. Scale bar in (B) and (C), 50 μm. DSGC, direction selective ganglion cells; DAPI, 4',6-diamidino-2-phenylindole; D, dorsal; V, ventral; N, nasal; T, temporal.

Retinas from New Zealand White rabbits aged P10-12 (around eye opening) were used to examine the distribution of the preferred directions of their DSGCs. A total of 27 animals were used in the present study. Nuclei of retinal ganglion cells were labeled with 4',6-diamidino-2-phenylindole (DAPI; Sigma, St. Louis, MO) 1–2 days before the experiment to allow the targeting of the DSGCs to be assessed. After anesthetizing the animals with intramuscular injection (IM) injection of ketamine (150 mg/kg) and xylazine (30 mg/kg), the eyes were treated with 0.5% proparacaine hydrochloride ophthalmic solution (Alcaine; Alcon-Couvreur, Belgium) before the intraocular injection of DAPI. On the day of the experiment, the animals were dark adapted for >1 h before dissection. After anesthetizing the animals with an IM injection of ketamine (300 mg/kg) and xylazine (60 mg/kg), 0.5% Alcaine was applied topically before enucleation under a dim red light. After hemisection, the lenses and vitreous humors were removed immediately. The posterior eyecups were then immersed in the oxygenated modified Ames's medium (120 mmol/L NaCl, 3.1 mmol/L KCl, 0.5 mmol/L KH_2_PO_4_, 1.2 mmol/L MgSO_4_, 1.15 mmol/L CaCl_2_, 6.0 mmol/L D-glucose) containing 23 mmol/L NaHCO_3_ (Sigma) (Ames and Nesbett 1981). Next, each retina was gently detached from the retinal pigment epithelium, and the peripheral region removed. The rabbits were then euthanized with CO_2_. The retinas were placed photoreceptor-side down and attached to a coverslip coated with the tissue adhesive (Cell-Tak; BD Biosciences, Bedford, MA). Each preparation was then transferred to a recording chamber mounted on the stage of a fluorescence microscope (Axioskop 2 FS Plus, Zeiss, Germany) and superfused with oxygenated modified Ames' medium (1.5∼2 mL/min) at 34–36°C. In order to map the preferred directions of DSGCs onto the canonical directions of animal axes (i.e., the dorsal–ventral axis and the nasal–temporal axis), the myelinated bands of retinas were always aligned horizontally in the recording chamber (Fig. 1A).

### Light stimuli

All visual stimuli were generated by VisionWorks (Vision Research Graphics, Durham, NH) and displayed on a CRT monitor (refresh rate 100 Hz; SyncMater 757NF; Samsung, Korea). The stimulus was reflected upward by a mirror positioned beneath the microscope stage. A 20× microscope objective (A-plan, NA 0.45, Zeiss) replaced the condenser and this was used to focus the stimulus onto the photoreceptor layer of the retina. The DSGCs were identified initially by their signature ON and OFF responses when they underwent flashing light stimulation, and subsequently by their direction selective responses when a bar of light was maneuvered manually. The preferred direction of the DSGCs was determined by a moving bar (540 × 180 μm^2^, ∼900 μm/sec) being swept across the receptive field center in 12 radial directions with equal spans. Luminance values on the stage ranged from <0.01 cd/m^2^ to 18 cd/m^2^ and these generally fell within the mesopic range.

### Extracellular recording

Retinal ganglion cells labeled with DAPI were visualized under brief fluorescence illumination (365 nm excitation) using a 40× water immersion objective (Achroplan, NA 0.8, Zeiss), and the DSGCs in the midperipheral region were targeted with the aid of nuclear features as described previously (Vaney 1994). A tungsten-in-glass electrode (∼1 MΩ) (Levick 1972) was used to record the spiking response of the ganglion cells, and the neural signal was amplified (1000×) and band-pass filtered (100Hz–3 kHz) using a differential amplifier (ISO-80; World Precision Instruments, Sarasota, FL). A LabVIEW based data acquisition system (10 kHz sampling rate; National Instruments, Austin, TX) was used to identify action potentials; furthermore, their time of occurrence relative to the stimulus generation was recorded by a computer for later offline analysis. After recording, the field of view was photographed with a digital camera (Coolpix P5100, Nikon, Melville, NY) to aid identification of the recorded cell (Fig. 1B).

### Intracellular dye injection

The tungsten-in-glass electrode was withdrawn after recording, and a micropipette with a filament (OD = 1.0 mm, ID = 0.5 mm; Sutter Instrument, Novato, CA) pulled by a programmable Flaming-Brown P97 puller (Sutter Instrument) was used to carry out intracellular dye injection. The dye solution was either 2% Lucifer Yellow (Sigma) and 4% Neurobiotin (Vector Laboratories, Burlingame, CA) in 0.1 mol/L Tris buffer or 10 mmol/L Alexa Fluor 555 (Molecular Probes, Eugene, OR) in 0.2 mol/L KCl. An intracellular amplifier (Neuroprobe Amplifier 1600, A-M Systems, Carlsborg, WA) was used to perform the iontophoresis at a current of −1 nA for 30 sec and 1∼2 nA for 1∼2 min for Lucifer Yellow/Neurobiotin injection, or −1∼−2 nA for 2 min for Alexa Fluor 555 injection. Immediately after dye injection, the targeted cell was photographed with a digital camera (Coolpix P5100, Nikon) to visualize its dendritic morphology (Fig. 1C). To allow diffusion of the Neurobiotin across the gap junctions, the tracer-filled cells were left in the oxygenated modified Ames' medium for at least 30 min prior to fixation in 4% paraformaldehyde (in 0.1 mol/L phosphate buffer [PB]) for 30 min. The Neurobiotin-injected cells were then visualized by incubating the retina with fluorescein isothiocyanate-conjugated streptavidin (diluted 1:50 in 0.1 mol/L PB with 0.1% Triton X-100; Vector Laboratories) at room temperature overnight. The retina was flat-mounted in the mounting medium (Vectashield; Vector Laboratories) for confocal imaging.

### Image acquisition

Images of the injected cells were acquired using a laser scanning confocal microscope (LSM 510, Zeiss) with a 20× objective lens (Plan-NEOFLUAR, NA 0.5, Zeiss). A series of z-stack images was taken from the focal plane of the axon fiber to the inner nuclear layer in order to reveal both the ON and OFF dendritic arbors of the DSGCs and their tracer-coupled cells. A LSM 510 image examiner (v4.0.0.241, Zeiss) was used to adjust the intensity and contrast of the image.

### Data analysis

Offline data analysis of the recorded extracellular spike trains was carried out using MATLAB (The MathWorks Inc, Natick, MA). To calculate the strength of the directional tuning, we used the direction selectivity index (DSI) (Taylor and Vaney 2002). Action potentials were recorded in each of 12 directions and the DSI was defined as:


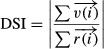


where *v*(*i*) is the vector pointing in the direction of the moving stimulus and having length *r*(*i*). The vector sum of the data from 12 directions points to the preferred direction of the DSGC. DSI can range from 0 (when the responses are equal across all 12 directions) to 1 (when the response is obtained only from a single direction). Thus, DSI values close to 1 indicate sharper directional tuning.

K-means clustering analysis (Seber 1984; Spath 1985) was used to evaluate the optimal classification scheme for distinguishing subtypes of DSGCs based on their preferred directions. All preferred directions were designated to have a length of 1 and these were transformed into Cartesian coordinates for subsequent distance measurement. This method optimizes the set of clusters with respect to the distance between each point and the centroid of its cluster, summed for all points. We chose 2–12 cluster numbers for comparison, and the fitness of clustering was evaluated using the silhouette value (SV) (Kaufman and Rousseeuw 1990).


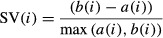


where *a*(*i*) is the average distance from point *i* to all other points in the same cluster, and *b*(*i*) is the average distance from point *i* to all points in a different cluster. A SV can range from 1 (perfectly clustered), through 0 (completely ambiguously clustered), to −1 (completely inappropriately clustered). Thus, SVs that are closer to 1 indicates better clustering. In addition, the histogram of preferred directions of DSGCs (bin = 5°) was analyzed using discrete Fourier transformation to calculate the angular periodicity.

All statistical analyses were performed using SPSS (SPSS Inc., Chicago, IL), and a value of *P* < 0.05 was considered significant. The preferred directions of the DSGCs from the two age groups were compared for each of four quadrants using the Student's *t*-test. The DSIs of the DSGCs from the two age groups were also compared using the Student's *t*-test. The DSIs in the four canonical sectors were compared using a general linear model. The correlation of preferred directions and DSIs in each of four quadrants was evaluated using Pearson's correlation.

## Results

### The distribution of the preferred directions of the DSGCs at around the time of eye opening cannot be distinctly segregated into four groups

**Figure 2:**
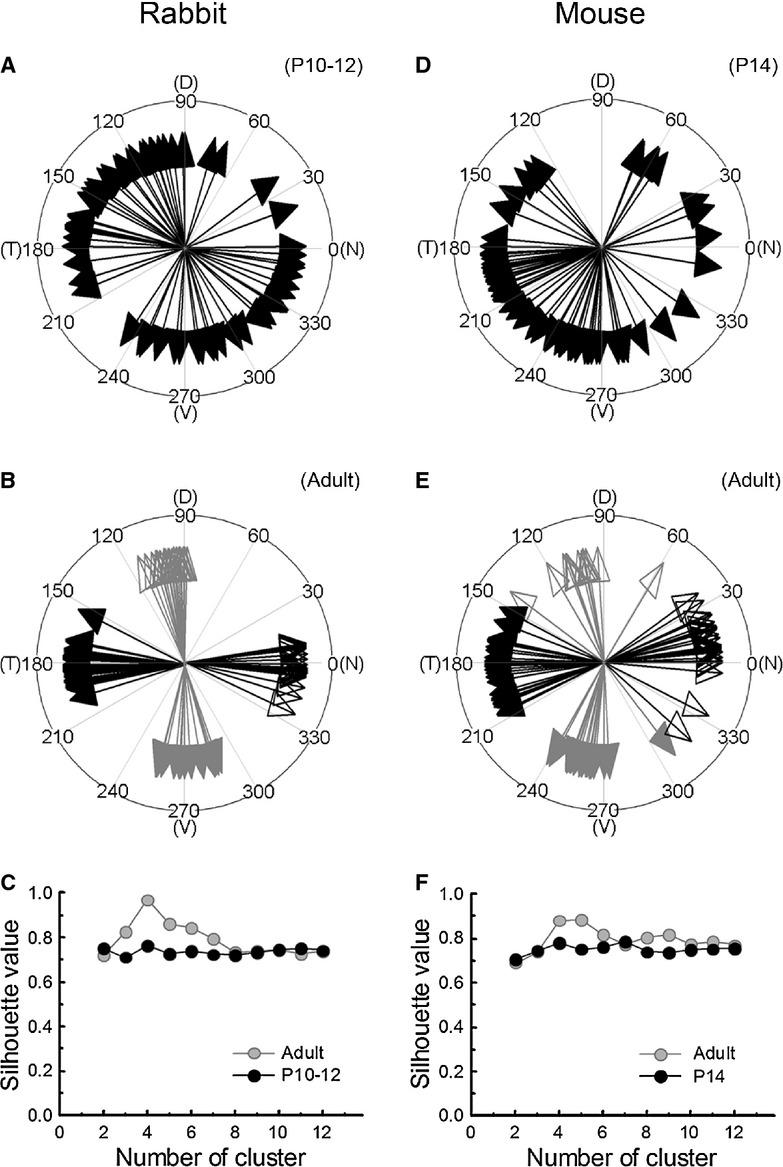
The distribution of preferred directions of DSGCs at around the time of eye opening cannot be separated into distinct clusters. (A) and (D) The preferred directions of the DSGCs in P10-12 rabbits and P14 mice were diffusely distributed (mouse data from Elstrott et al. 2008). (B) and (E) The distribution of preferred directions of DSGCs in adult rabbits and mice can be classified into four canonical directions, depicting by the open/closed and black/gray arrows, which are based on the k-means cluster analysis (rabbit data from Oyster 1968; mouse data from Elstrott et al. 2008). (C) and (F) Average silhouette values of the different cluster numbers were calculated using k-means cluster analysis for the distribution of the preferred directions of the DSGCs in adult and postnatal rabbits and mice. DSGC, direction selective ganglion cells; D, dorsal; V, ventral; N, nasal; T, temporal.

It is known that DSGCs in the adult rabbit retina can be classified into four subtypes based on the distribution of their preferred directions (Oyster and Barlow 1967; Oyster 1968). However, we found that the preferred directions of some DSGCs cannot be assigned to any one of the four canonical directions, that is, the dorsal, ventral, nasal, and temporal retinal axes that correspond to the inferior, superior, posterior, and anterior axes of the visual field (one example in Fig. 1D; all 64 cells in Fig. 2A). Further analysis using k-means clustering supports this observation, in that the average SVs were found to be similar across all different cluster numbers (Fig. 2C). This suggests that no specific cluster numbers best classify the distribution pattern of preferred directions of DSGCs in P10-12 rabbit retinas. To compare this finding with the distribution of preferred directions of DSGCs in adult rabbit retinas, we also used k-means clustering analysis to examine their distribution pattern (Fig. 2B; *n* = 92; data from Oyster 1968). It is apparent that the average SV was much higher for four specific clusters (0.97) than for any of the other cluster numbers (Fig. 2C). This indicates that the distribution of preferred directions of the DSGCs in adult rabbits can be reliably segregated into four distinct groups.

In the mouse retina, Elstrott et al. (2008) reported that the preferred directions of DSGCs in both adult and P14 animals (at around the time of eye opening) can be separated into four groups and corresponded to the four canonical axes. To compare their findings with ours, we again used k-means clustering analysis to examine the distribution of preferred directions of the DSGCs obtained from mouse retinas (Fig. 2D and E; *n* = 74 and 90; data from Elstrott et al. 2008). Similar to the result obtained from adult rabbits, the average SV of the DSGCs in adult mice was higher for the four clusters (0.88) than for most other cluster numbers (Fig. 2F). Although the average SV was also high for five clusters, this appeared to be a poor and unacceptable result, because there were only three cells in one of the clusters. Interestingly, the clustering result for the P14 mice was also similar to our findings for the P10-12 rabbits. There was no peak of average SVs across all different cluster numbers in the P14 mice (Fig. 2F). This contrasts with the conclusion of Elstrott et al. (2008) and ours analysis suggests that the preferred directions of DSGCs in mice at around the time of eye opening cannot be segregated into four distinct groups just like the results for rabbits.

### The preferred directions of DSGCs are diffusely distributed along the four canonical axes immediately after eye opening

**Figure 3:**
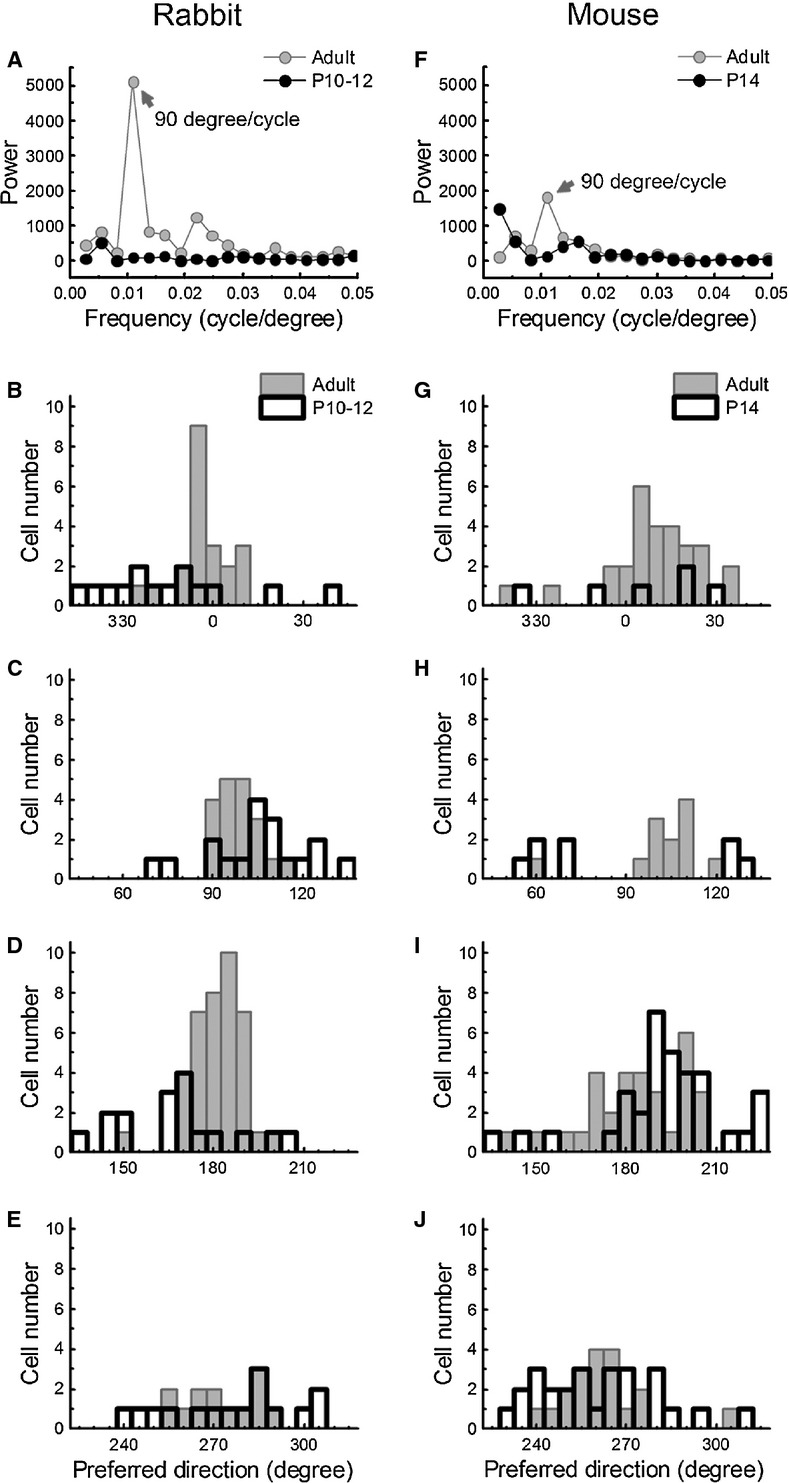
The preferred directions of the direction selective ganglion cells (DSGCs) at around the time of eye opening are diffusely distributed across the four canonical axes. (A) and (F) Periodicity analyses of the preferred direction distributions of the DSGCs in adult and postnatal rabbits and mice (adult rabbit data from Oyster 1968; all mouse data from Elstrott et al. 2008). Based on the Fourier power spectra, the periods of the preferred direction distributions of the DSGCs in adult animals were ∼90°, whereas those of the DSGCs in the postnatal animals showed no apparent periodicity. (B–E) and (G–J) Histograms of the preferred directions of the DSGCs across each of four canonical axes (0, 90, 180, and 270°) in adult and postnatal rabbits and mice.

To further characterize the distributions of preferred directions of the DSGCs in adult and P10-12 rabbits, the angular periodicity of the preferred directions was calculated using Fourier transformation. In P10-12 rabbit retinas, there was no apparent periodicity for the distributions of the preferred directions of the DSGCs (Fig. 3A), which is consistent with the results from the clustering analysis (Fig. 2C). However, an angular periodicity of ∼90° was observed for the distributions of preferred directions of DSGCs in adult rabbit retinas (Fig. 3A). This result implies that in adult rabbits there are four clusters for the preferred directions of the DSGCs and these are organized along the four orthogonal axes.

To examine if these four orthogonally orientated groups of DSGCs are aligned in canonical axes of the retinal field (dorsal, ventral, nasal, and temporal), the preferred directions of the DSGCs in four quadrants centered on the canonical axes (0, 90, 180, and 270°) were separately plotted (Fig. 3B–E). In general, the histograms in each quadrant were much broader for the P10-12 retinas than for the adult retinas, which suggests that the preferred directions of the DSGCs at around the time of eye opening are more diffusely distributed along the four canonical axes than those of adult animals. Despite this noticeable difference, the average preferred directions of the DSGCs in each canonical axis were not significantly different between adult and P10-12 retinas, except for the temporal axis (Table 1). However, the standard deviations of the preferred directions of the DSGCs in each quadrant were much larger for the P10-12 retinas than for the adult retinas (Table 1). This supports the preferred directions of DSGCs immediately after eye opening are not narrowly distributed.

**Table 1:** The statistics for the preferred directions of DSGCs along each of four canonical axes in adult and postnatal rabbits and mice

Region	Nasal		Dorsal		Temporal		Ventral	
Rabbit								
Stage	Adult	P10-12	Adult	P10-12	Adult	P10-12	Adult	P10-12
Cell number	21	14	19	18	39	17	13	15
Mean (degree)	356.57	346.40	98.45	105.39	181.17	166.92	271.99	275.25
SD (degree)	8.91	23.39	6.57	16.79	8.61	18.69	11.43	20.96
Student's *t-*test (*P*)	0.141		0.116		0.007		0.608	
Mouse								
Stage	Adult	P14	Adult	P14	Adult	P14	Adult	P14
Cell number	28	6	12	8	31	34	19	26
Mean (degree)	369.09	365.67	101.23	87.60	183.49	192.53	261.40	260.09
SD (degree)	16.34	24.21	14.55	33.00	16.39	19.42	14.13	19.14
Student's *t*-test (*P*)	0.672		0.301		0.048		0.802	

Adult rabbit data from Oyster 1968; all mouse data from Elstrott et al. 2008.

Similarly, we also calculated the angular periodicity of the preferred directions of the DSGCs in adult and P14 mice using the data from Elstrott et al. (2008). In the P14 mouse retinas, there was no apparent periodicity for the distributions of the preferred directions of the DSGCs (Fig. 3F), which is consistent with the result of clustering analysis (Fig. 2F). However, an angular periodicity of ∼90° was observed for the distributions of the preferred directions of the DSGCs in adult mouse retinas (Fig. 3F). This result corroborates that there are four orthogonally organized subtypes of DSGCs in adult mouse retinas (Fig. 2F). When separately plotting the preferred directions of the DSGCs in the four quadrants centered on the canonical axes of the adult and P14 mouse retinas, similar results to the adult and P10-12 rabbits were observed (Fig. 3G–J). The histograms in each quadrant were much broader for P14 retinas than for adult retinas. This observation is supported by the fact that the standard deviations of preferred directions of DSGCs in each quadrant were much larger in P14 retinas than in adult retinas (Table 1). These findings indicate that the preferred directions of DSGCs of the P14 mice at around the time of eye opening are more diffusely distributed along the four canonical axes than those of the adult animals.

### The distribution of the preferred directions of the DSGCs at around the time of eye opening does not correlate with their direction tuning strength

In the mouse retina, Elstrott et al. (2008) reported that the direction tuning strength of DSGCs at P14 in mice was weaker than that of the same cells in the adult, although Chen et al. (2009) found that the tuning width and DSI (and the variability) of P11, P13, P18, and adult DSGCs were very similar. In the rabbit retina, we have also observed that the DSIs of DSGCs showed a greater variation in P10-14 rabbits than in adults (Chan and Chiao 2008). To compare the direction tuning strength of DSGCs at around the time of eye opening (P10-12) and that of the same cells in adult rabbits (Chan and Chiao 2008), we pooled the DSIs of DSGCs of the P10-12 rabbits from the present study with those from our previous study. Similar to the observations of Elstrott et al. (2008), we also found that the DSIs in the P10-12 retinas were significantly lower than those in adult retinas *(P* = 0.013, Table 2). This suggests that the direction tuning strength of DSGCs is still developing at the time of eye opening in mammalian retinas.

**Table 2:** A comparison of the tuning strengths of DSGCs in adult and postnatal rabbits

DSI				

Stage	Cell number	Mean	SD	Student's *t*-test (*P*)
Adult	12	0.51	0.14	0.013
P10-12	73	0.40	0.14	

Adult data from Chan and Chiao 2008; P10-12 data from Chan and Chiao 2008 (*n* = 9) and the present study (*n* = 64).

**Figure 4:**
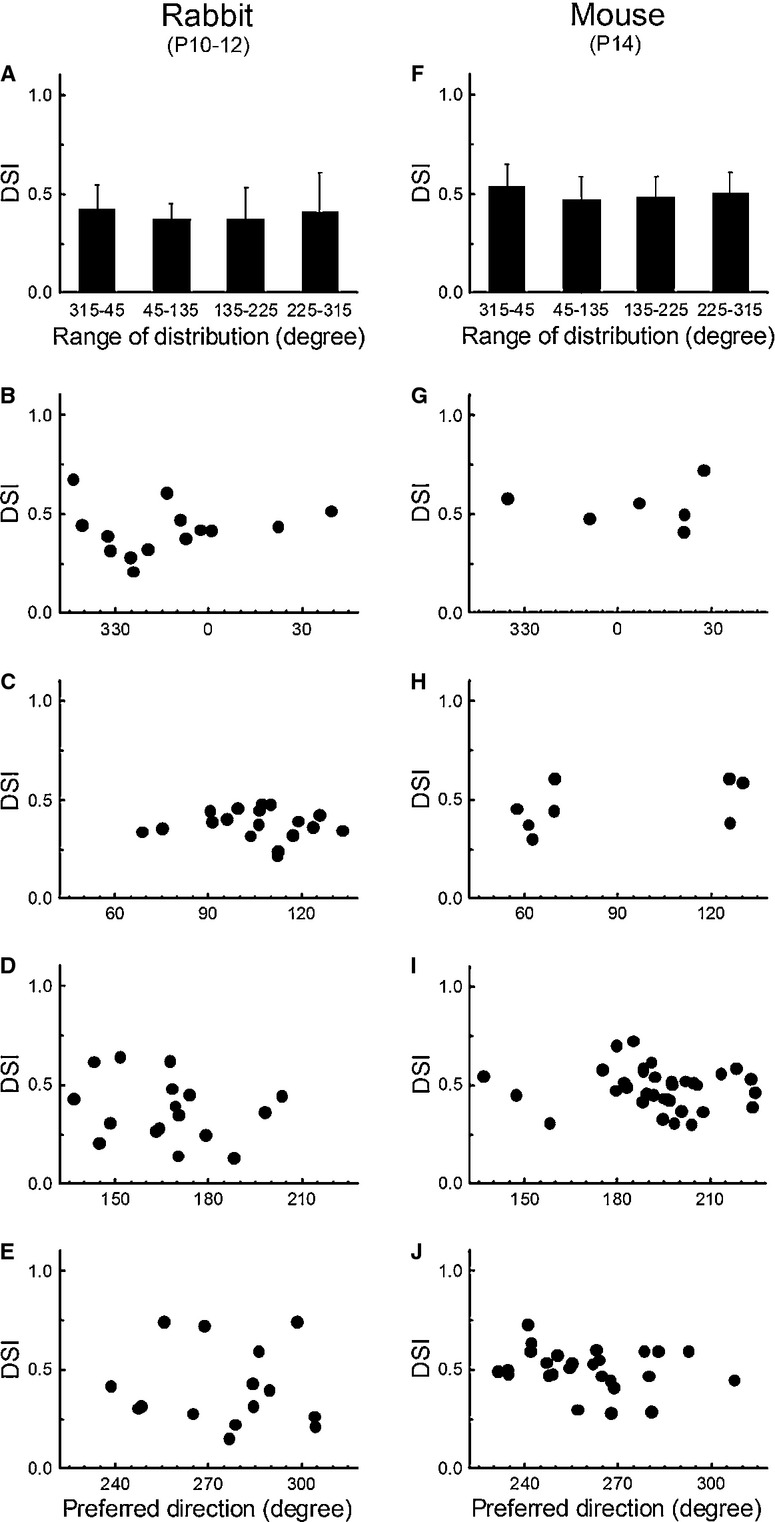
The preferred direction distributions of the direction selective ganglion cells (DSGCs) at around the time of eye opening are independent of the tuning strength of their direction selectivity. (A) and (F) Average direction selectivity indexes (DSIs) of the DSGCs whose preferred directions fell within the ranges of four canonical axes (0, 90, 180, and 270°) in postnatal rabbits and mice (mouse data from Elstrott et al. 2008). Error bars indicate SD. (B–E) and (G–J) Scatter plots of the DSIs of the DSGCs whose preferred directions are distributed across each of four canonical axes in postnatal rabbits and mice.

To analyze if the direction tuning strengths of DSGCs at around the time of eye opening in rabbit retina are differentially increased across the different canonical axes, the average DSIs of the DSGCs in four quadrants were compared (Fig. 4A). We found that there was no significant difference in DSIs among the four subgroups of DSGCs (*P* = 0.77), which suggests that the direction tuning strengths of DSGCs in the four quadrants are at similar levels at eye opening. To further examine if the direction tuning strengths of the DSGCs are higher for those cells whose preferred directions are closely aligned with four canonical axes, we plotted the DSIs of DSGCs in each quadrant as a function of their preferred directions (Fig. 4B–E). We found that there was no correlation between the DSIs and the deviation of preferred directions from the canonical axes across all four quadrants (*R* = 0.18, −0.27, 0.30, and −0.10; *P* = 0.55, 0.28, 0.25, and 0.73). This suggests that the preferred directions of DSGCs are independent of their direction tuning strengths at around the time of eye opening in the rabbit retina.

Similarly, we compared the average DSIs of the DSGCs across the four quadrants of the P14 mice using the data from Elstrott et al. (2008), and found that there was no significant difference in DSIs across the four subgroups of DSGCs (*P* = 0.58; Fig. 4F). In addition, we also found that there was no correlation between the DSIs and their preferred directions across all four quadrants (Fig. 4G–J; *R* = 0.39, 0.15, −0.22, 0.30; *P* = 0.44, 0.73, 0.20, 0.13). This result indicates that the direction tuning strengths of the DSGCs in P14 mice are independent of their preferred directions in the developing mouse retinas, and that they are not correlated with the proximity of their preferred directions to the canonical axes.

### The preferred directions of DSGCs displaying tracer coupling patterns immediately after eye opening do not conform to one canonical direction

**Figure 5:**
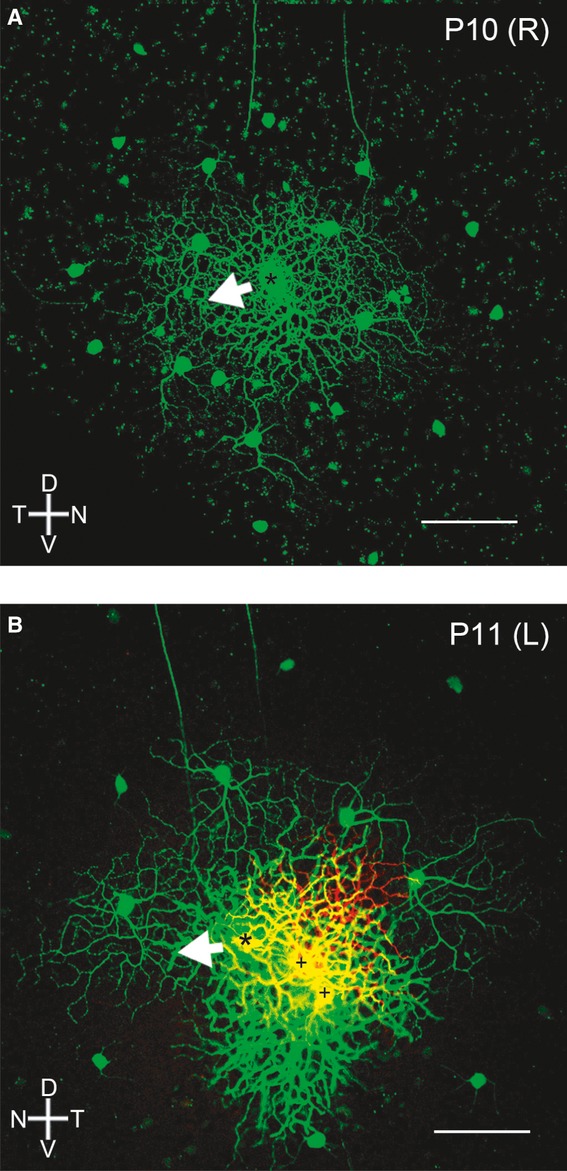
The direction selective ganglion cells (DSGCs) with prominent tracer coupling patterns at around the time of eye opening do not have the same preferred directions. Confocal micrographs showing DSGCs injected with Neurobiotin (asterisks) and their tracer coupling patterns (green) after electrophysiological recordings. (A) The preferred direction of the DSGC in the right eye of a P10 rabbit was toward its temporal axis. (B) The preferred direction of the DSGC in the left eye of a P11 rabbit was toward its nasal axis. The plus signs are two other recorded DSGCs injected with Alexa555 (red) and Neurobiotin (green), one with a preferred direction toward its dorsal axis (red) and the other with a preferred direction toward its temporal axis (green). Scale bar, 100 μm.

**Figure 6:**
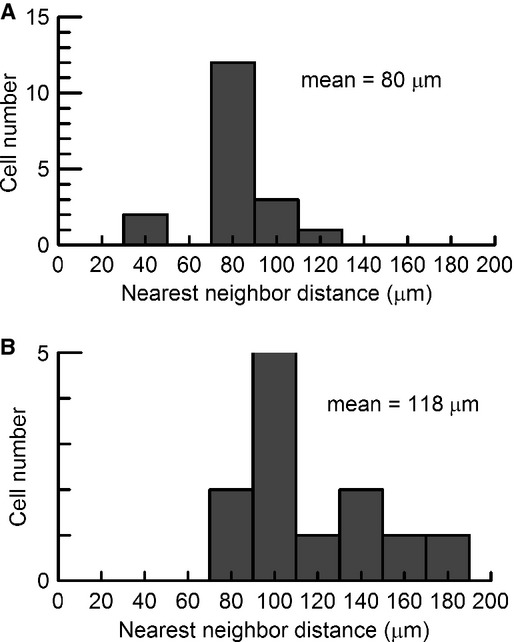
The nearest neighbor analysis of tracer coupled DSGCs shown in Figure 5. (A) The DSGC and its 17 coupled cells shown in Figure 5A had an average nearest neighbor distance of 80 ± 4 *μ*m. (B) The DSGC and its 11 coupled cells shown in Figure 5B had an average nearest neighbor distance of 118 ± 9 *μ*m. The apparent exclusion zone of the nearest neighbor distance histogram suggests that the coupling is homologous in DSGCs. Note that the gap in the histogram of (A) was due to small sampling size.

**Figure 7:**
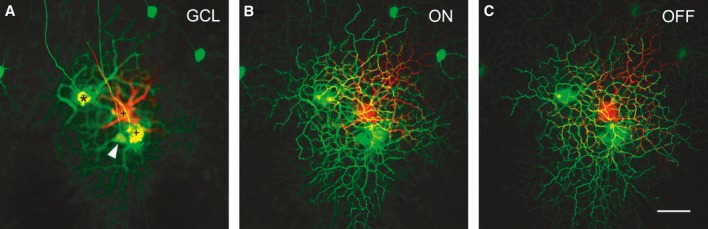
Confocal images of DSGCs at different focal planes shown in Figure 5B. (A) The somata and axons of Neurobiotin-injected DSGC (asterisk) its coupled cells (green) in the ganglion cell layer (GCL). The plus signs are two other recorded DSGCs injected with Alexa555 (red) and Neurobiotin (green). Note that a cell (arrowhead) coupled to the asterisk cell was next to one of the Neurobiotin injected cells (plus, green). The fact that this coupled cell (arrowhead) was next to the Neurobiotin injected cell (plus, green) argues against the direct tracer coupling between these two cells. (B and C) The ON arbors and OFF arbors of three injected DSGCs in (A) in the inner plexiform layer. Note that the red cell was not coupled to both green cells. Scale bar, 50 *μ*m.

To examine the preferred directions of DSGCs and their corresponding tracer coupling patterns at around the time of eye opening in developing rabbit retinas, Neurobiotin was injected into the DSGCs after determining their preferred directions. Despite the technical difficulties, two DSGCs that showed extensive tracer coupling patterns were found in the P10-12 retinas. Unlike the adult rabbit retina, in which only the ventral (or superior) subtype shows tracer coupling patterns (Kanjhan and Vaney 2008), the preferred directions of these two tracer-coupled DSGCs were temporal and nasal subtypes (Fig. 5). The DSGC with the temporal preferred direction in a P10 rabbit had their tracer-coupled cells outside the dendritic field, suggesting that they are the same subtype (Fig. 5A). The nearest neighbor analysis also supports that the coupling was homologous (Fig. 6A). The DSGC with the nasal preferred direction in a P11 rabbit also had a similar pattern of tracer coupling (Fig. 5B). In the same field, another two recorded DSGCs were injected as well. One DSGC filled with Alexa555 (red) had a dorsal preferred direction, and the other DSGC filled with Lucifer Yellow/Neurobiotin (green) had a temporal preferred direction. Although these two DSGCs were injected with Neurobiotin, the characteristic pattern of concentric tracer coupling suggests that the DSGC with the nasal preferred direction was the DSGC displaying the tracer coupling pattern (see Fig. 7 for different views). The nearest neighbor analysis also confirms that the coupling was homologous (Fig. 6B). Nevertheless, this result indicates that the preferred directions of the DSGCs with tracer coupling patterns in P10-12 rabbit retinas are not the same as those in adult retinas, and they do not conform to one canonical direction.

## Discussion

We have demonstrated that the preferred directions of the four subtypes of DSGCs are diffusely distributed along each of four canonical axes at the time of eye opening in the rabbit retina. Unlike the previous findings for the adult rabbit retina (Oyster and Barlow 1967; Oyster 1968), immediately after eye opening the four subtypes of DSGCs cannot be segregated into four distinct groups based on their preferred directions. We have also demonstrated that the diffuse pattern of preferred direction distribution does not correlate with the directional tuning strength of the DSGCs, indicating that the maturation of direction selectivity and preferred direction are independent processes. Moreover, we have provided evidence that argues against the hypothesis that only one subtype of DSGCs is tracer coupled at the time of eye opening (Vaney 1994; DeBoer and Vaney 2005; Kanjhan and Vaney 2008; Vaney et al. 2012). Taken together, our finding indicates that four subtypes of DSGCs, as they develop from eye opening to adulthood, undergo significant refinement with respect to their preferred directions and tracer coupling patterns.

### The four subtypes of DSGCs continue to refine after eye opening

The current understanding of DSGC development suggests that the directional circuitry is established shortly before eye opening in the mammalian retina (Bowe-Anders et al. 1975; Masland 1977; Chan and Chiao 2008; Elstrott et al. 2008; Chen et al. 2009). More recent studies have also supported this view by demonstrating that development of spatially asymmetric inhibition from starburst amacrine cells onto the DSGCs is complete before eye opening (Wei et al. 2011; Yonehara et al. 2011). Furthermore, it has been hypothesized that the four subtypes of the DSGCs establish their adult pattern of preferred direction distribution at around the time of eye opening (Elstrott et al. 2008; Sun et al. 2011). However, the present study demonstrated unequivocally that DSGCs have not reach their final functional organization at moment of eye opening, but rather they continue to refine after eye opening.

**Figure 8:**
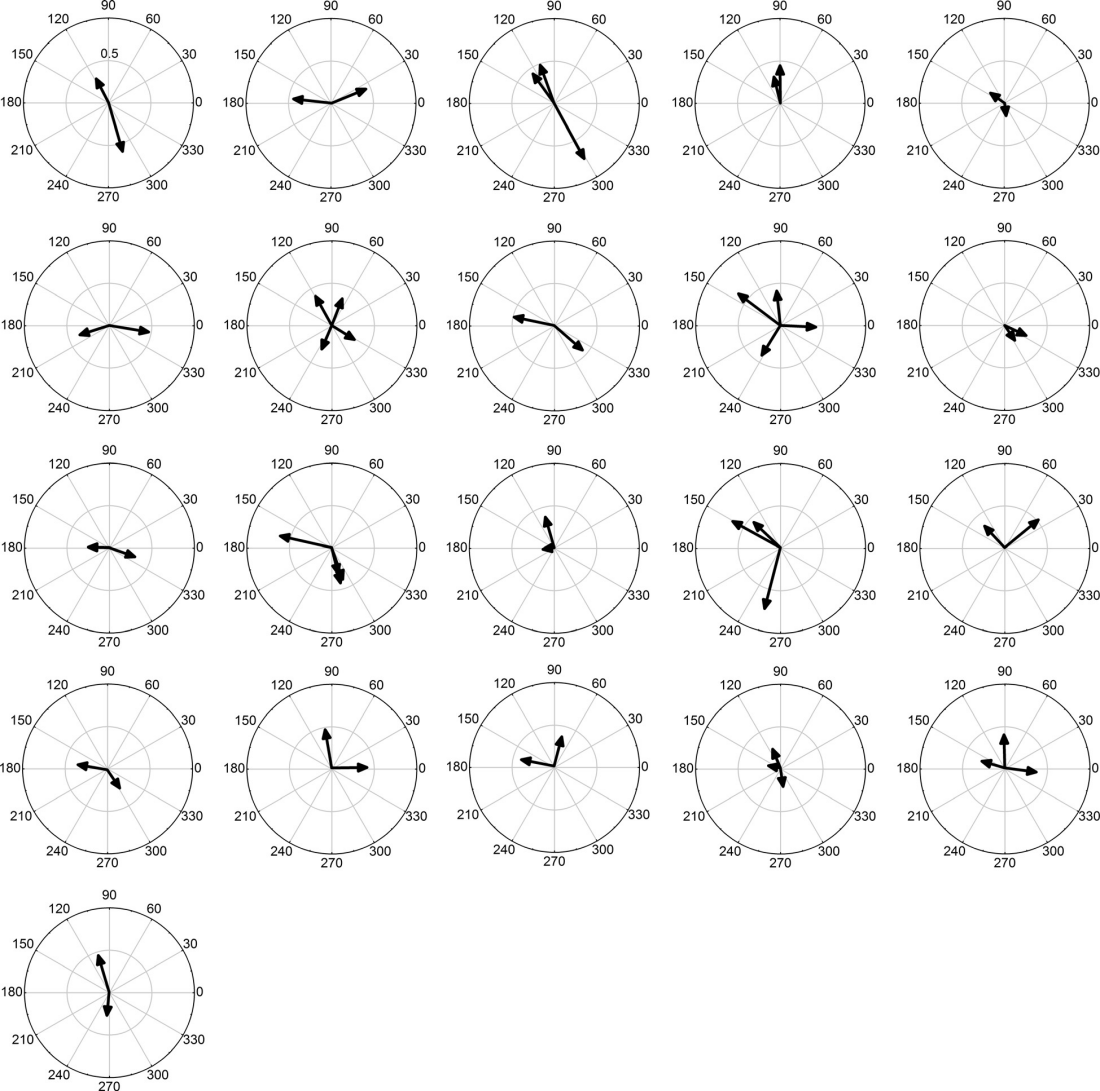
The preferred directions of 2–4 DSGCs recorded on the same retina around the time of eye opening are not perfectly aligned along the canonical axes. There were 21 retinas from which more than one DSGC was recorded, and their preferred directions were shown in the polar plots for individual retinas. The vector length indicates the DSI of each cell.

**Figure 9:**
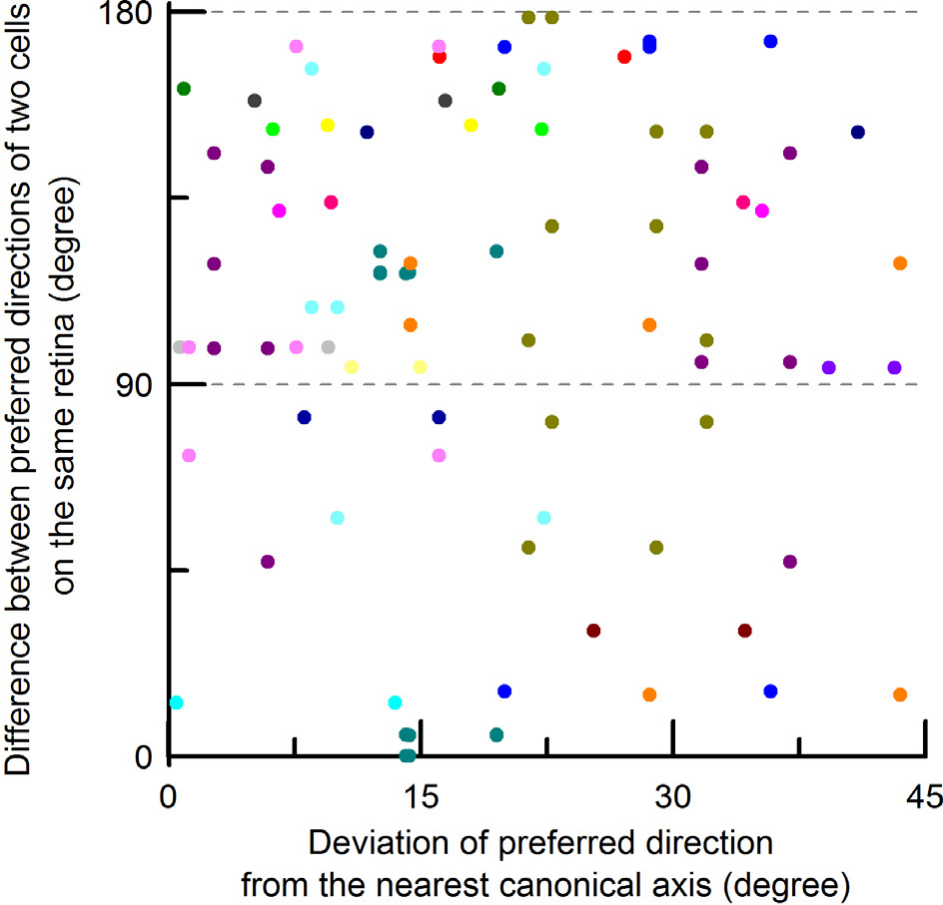
The preferred directions of DSGCs on the same retina at around the time of eye opening are not aligned orthogonally or in opposite directions. The difference between preferred directions of two DSGCs on the same retina was plotted against the deviation of preferred direction of one of the DSGCs from its nearest canonical axis. The lower and upper dashed gray lines indicate the perfect orthogonal and opposite differences, respectively. Different colors are cells recorded from different retinas. The fact that most differences of preferred directions between two cells on the same retina were not distributed along two gray dashed lines suggests that the dispersion of the preferred direction observed in Figure 2A is not caused by handling and placing the retina in the recording chamber.

Although previous studies have shown that all four subtypes of DSGCs in the adult rat and mouse retinas can be recorded at around the time of eye opening (Elstrott et al. 2008; Sun et al. 2011), these studies have been either based on a small number of recorded cells or have used a less sophisticated analysis of the preferred direction distribution. As such, these studies may have overlooked the establishment of the four distinct subtypes of DSGCs at eye opening. To characterize the distribution pattern of the DSGC's preferred directions at a finer level, we applied several computational methods to analyze the preferred directions of the DSGCs from P10-12 rabbits (the present study) and P14 mice (data from Elstrott et al. 2008). Our results showed clearly that the four subtypes of DSGCs do not display their adult patterns of preferred direction distribution at around the time of eye opening (Figs. 2, 3, Table 1). To rule out the possibility that the dispersion of the preferred directions in P10-12 rabbits observed in Figure 2A was resulted from mishandling or misplacing the retina in the recording chamber, we performed the pairwise analysis for cells recorded from the same retina and examined if their preferred direction differences fall into the category of 90 or 180°. Our results showed that the preferred directions of DSGCs on the same retina are not aligned orthogonally or in opposite directions at around the time of eye opening (Figs. 8 and 9). This supports that the dispersion of the preferred directions in P10-12 rabbits was not caused by handling and placing the retina during the experiments. Taken together, these findings suggest that the refinement of the DSGC's preferred directions after eye opening may be a general feature during the development of many if not all mammalian retinas.

It has been reported that the strength of the directional tuning of DSGCs is weaker at around the time of eye opening in the mouse retina (Elstrott et al. 2008), with an exception (Chen et al. 2009). Although the present study also showed a similar trend for the rabbit retina (Table 2), no correlation was found between the tuning strengths of the DSGCs and their preferred directions (Fig. 4). In a different study, it has been found that the dopamine receptor 4 (DRD4) and thyrotropin-releasing hormone receptor (TRHR) genetic mouse lines both labeled the nasal subtype of DSGCs and had similar preferred direction distributions (Huberman et al. 2009; Rivlin-Etzion et al. 2011), but cells in the TRHR line showed weaker directional tuning strength than those in the DRD4 line (Rivlin-Etzion et al. 2011). These findings also suggest that there is no apparent correlation between the tuning strengths of DSGCs and their preferred directions. Taken together, the above evidence as a whole supports the hypothesis that the direction selectivity of DSGCs and their preferred direction refinement occur via different developmental processes in order to attain their adult forms.

In addition to the preferred direction refinement, it has been proposed that DSGCs undergo a transition from all four subtypes electrically coupled together to only the ventral subtype coupled with each other in the developing rabbit retina (Vaney 1994; DeBoer and Vaney 2005; Kanjhan and Vaney 2008). This type of tracer coupling refinement suggests that there is decoupling of the DSGCs during development and this contributes in part to the establishment of the direction selectivity circuitry (DeBoer and Vaney 2005). Although our result demonstrates that both nasal and temporal subtypes of DSGCs were still tracer coupled at P10-12, the adult pattern of tracer coupling (restricted to only the ventral subtype) might be reached either sequentially or simultaneously soon after eye opening (Vaney 1994). This also supports the hypothesis that all four subtypes of DSGCs indeed continue to undergo refinement of the neural circuitry after eye opening.

### Refinement of preferred directions of DSGCs but not their subtype induction is activity dependent

Recent studies of transgenic mice have identified several distinct subtypes of DSGCs based on their genetic profiles (Huberman et al. 2009; Kim et al. 2010; Kay et al. 2011; Rivlin-Etzion et al. 2011; Trenholm et al. 2011). A recent study has shown that only multipotential progenitors expressing cadherin 6 determine the dorsal and ventral subtypes of DSGCs that respond selectively to the vertical direction (De la Huerta et al. 2012). These findings support the idea that the establishment of subtype identity of DSGCs before eye opening is a genetically specified process (Elstrott and Feller 2009; Vaney et al. 2012).

Despite the importance of initial subtype induction, the maturation of functional DSGCs may still require significant refinement. It is well recognized that activity-dependent refinement is one of general features that occur during the establishment of functional neural circuits (Lu et al. 2009; Sanes and Yamagata 2009; Bleckert and Wong 2011). For example, retinal waves and visual activity are known to contribute to refinement of the retinogeniculate circuitry (Hooks and Chen 2006, 2008; Hong and Chen 2011). In the ferret visual cortex, it has also been found that the development of direction selectivity requires visual activity (Li et al. 2006, 2008), although direction selectivity seems to develop normally in the visual cortex of dark-reared mice (Rochefort et al. 2011). While it has been confirmed that the initial establishment of direction selectivity circuitry in the retina is independent of retinal waves and visual activity (Chan and Chiao 2008; Elstrott et al. 2008; Chen et al. 2009; Sun et al. 2011), the present study indicates that the four subtypes of DSGCs have not achieve their adult pattern of preferred directions at around the time of eye opening. This argues in support of the hypothesis that refinement of the direction selectivity circuitry is dependent on visual activity after eye opening.

A careful examination has revealed that DSGCs in the P14 mouse retina are not distributed equally across all four quadrants, but rather the dorsal and nasal subtypes of DSGCs are outnumbered by the ventral and temporal subtypes (Elstrott et al. 2008). However, the four subtypes of DSGCs have roughly equal numbers in the adult mouse retina (Elstrott et al. 2008). This apparent mismatch in the numbers of DSGC subtypes in the developing mouse retina is reminiscent of a recent discovery whereby the development of direction selectivity in mouse cortical neurons at the time of eye opening is also dominated by the dorsal and nasal subtypes (correspond to ventral and temporal axes in the retina) (Rochefort et al. 2011). Interestingly, it has been found that retinal waves have an overall preference of propagating toward the dorsal–nasal retina in the in vivo P3-9 mouse superior colliculus and primary visual cortex (Ackman et al. 2012), as well as the in vitro mouse retina (Stafford et al. 2009; Elstrott and Feller 2010). Although the propagation of these retinal waves is directly opposite to the direction preference of the DSGCs in the retina and visual cortex, this wave direction bias may mediate some aspects of the early development of the direction selectivity circuitry. The fact that the numbers of the four subtypes of DSGCs are equal in the adult mouse retina also suggests that visual activity may play a role in refining their preferred directions and in adjusting the numbers after eye opening.

In a sharp contrast to the above results, a mismatch in the numbers of DSGC subtypes was found in the adult rabbit retina (Oyster and Barlow 1967; Oyster 1968), but not in the P10-12 retina (Fig. 3). It is not known whether the propagation of the retinal waves has any directional preference in the rabbit retina (Syed et al. 2004) Thus, unlike in the mouse retina, DSGCs in the rabbit retina may have developed equal numbers of the four subtypes at eye opening, and these cells gradually refine their preferred directions and numbers in a visual activity-dependent manner in order to attain their adult form. This hypothesis is partially supported by a classic experiment in which exposing dark-reared rabbits briefly each day to a unidirectionally moving environment significantly altered the cell population of the four subtypes of DSGCs and this resulted in their distribution of preferred directions becoming more diffuse, although the DSGCs still developed into four subtypes normally (Daw and Wyatt 1974).

### Implications of the DSGC preferred direction distribution at eye opening

The four preferred directions of the DSGCs appear to be aligned with the four rectus muscles (Oyster and Barlow 1967; Oyster 1968). Furthermore, the four preferred directions of the DSGCs are projected onto the nucleus of the optic tract (Pu and Amthor 1990), which is reciprocally connected with the nuclei of the accessory optic system (Simpson 1984). Finally, the optokinetic reflex is abolished after elimination of direction selectivity in the retina (Yoshida et al. 2001; Amthor et al. 2002). These findings all suggest that the output of the DSGCs is responsible for controlling eye movement in the rabbit (Oyster 1968). Our observation that the preferred directions of four subtypes of DSGCs are diffusely distributed across each of four canonical axes at around the time of eye opening implies that the optokinetic reflex is not fully functional immediately after eye opening. This is supported by evidence showing that the optokinetic reflex is not observable until 3 weeks after birth in rabbits and mice (Daw and Wyatt 1974; Faulstich et al. 2004). In addition, it is known that visual experience is necessary for the establishment of the ocular motor system and the development of the extraocular muscles (McMullen et al. 2004). It would be of great interest to examine if the refinement of the DSGC preferred directions coincides with the functional maturation of optokinetic reflex after eye opening in rabbits and mice.

In addition to controlling eye movement, the projection of the DSGCs onto the LGN also suggests that this projection has a role in the image-forming pathway (Levick et al. 1969; Piscopo et al. 2013). In the LGN, it has been hypothesized that the sharp tuning of direction selective neurons is a result of excitation and inhibition from spatially superimposed mirror-symmetric subtypes (dorsal/ventral or temporal/nasal) (Levick et al. 1969; Montero and Brugge 1969; Marshel et al. 2012). Our observation that the preferred directions of the four subtypes of DSGCs are diffusely distributed along each of four canonical axes at around the time of eye opening also implies that there is sharpening of the direction selective neurons in the LGN and these neurons have not attained their adult level of selectivity immediately after eye opening. This implies that directional information relating to moving objects in the rabbit will be less accurate at eye opening. As the LGN is a relay station for information transmission to the visual cortex, this late maturation of the direction selective neurons in the LGN may have a profound influence on the development of the visual cortex.
